# Comparative Analyses of SUV420H1 Isoforms and SUV420H2 Reveal Differences in Their Cellular Localization and Effects on Myogenic Differentiation

**DOI:** 10.1371/journal.pone.0014447

**Published:** 2010-12-29

**Authors:** Leanna W. K. Tsang, Ninghe Hu, D. Alan Underhill

**Affiliations:** 1 Department of Medical Genetics, School of Human Development, University of Alberta, Edmonton, Alberta, Canada; 2 Department of Oncology, School of Cancer, Engineering, and Imaging Sciences, Faculty of Medicine and Dentistry, University of Alberta, Edmonton, Alberta, Canada; George Mason University, United States of America

## Abstract

**Background:**

Methylation of histone H4 on lysine 20 plays critical roles in chromatin structure and function via mono- (H4K20me1), di- (H4K20me2), and trimethyl (H4K20me3) derivatives. In previous analyses of histone methylation dynamics in mid-gestation mouse embryos, we documented marked changes in H4K20 methylation during cell differentiation. These changes were particularly robust during myogenesis, both in vivo and in cell culture, where we observed a transition from H4K20me1 to H4K20me3. To assess the significance of this change, we used a gain-of-function strategy involving the lysine methyltransferases SUV420H1 and SUV420H2, which catalyze H4K20me2 and H4K20me3. At the same time, we characterized a second isoform of SUV420H1 (designated SUV420H1_i2) and compared the activity of all three SUV420H proteins with regard to localization and H4K20 methylation.

**Principal Findings:**

Immunofluorescence revealed that exogenous SUV420H1_i2 was distributed throughout the cell, while a substantial portion of SUV420H1_i1 and SUV420H2 displayed the expected association with constitutive heterochromatin. Moreover, SUV420H1_i2 distribution was unaffected by co-expression of heterochromatin protein-1α, which increased the targeting of SUV420H1_i1 and SUV420H2 to regions of pericentromeric heterochromatin. Consistent with their distributions, SUV420H1_i2 caused an increase in H4K20me3 levels throughout the nucleus, whereas SUV420H1_i1 and SUV420H2 facilitated an increase in pericentric H4K20me3. Striking differences continued when the SUV420H proteins were tested in the C2C12 myogenic model system. Specifically, although SUV420H1_i2 induced precocious appearance of the differentiation marker Myogenin in the presence of mitogens, only SUV420H2 maintained a Myogenin-enriched population over the course of differentiation. Paradoxically, SUV420H1_i1 could not be expressed in C2C12 cells, which suggests it is under post-transcriptional or post-translational control.

**Conclusions:**

These data indicate that SUV420H proteins differ substantially in their localization and activity. Importantly, SUV420H2 can induce a transition from H4K20me1 to H4K20me3 in regions of constitutive heterochromatin that is sufficient to enhance myogenic differentiation, suggesting it can act an as epigenetic ‘switch’ in this process.

## Introduction

An essential component of chromatin biology involves the post-translational modification of the core histones H2A, H2B, H3 and H4. This can involve the addition of acetyl, methyl, phosphate, ADP-ribosyl, or ubiquitin moieties to over 30 target sites, which is further extended by the placement of multiple methyl groups on lysine and arginine [1], [2], [3], [4]. In the case of lysine methylation, this involves the transfer of up to three methyl groups from S-adenosyl-methionine to create mono-, di-, and trimethyl derivatives [5], [6]. These lysine modifications are limited to histones H3, where they can occur on multiple residues, and H4, which contains a single target site at position 20 (H4K20) [6]. In metazoans, the formation of H4K20me1 is catalyzed by PR-Set7/KMT5A [7], [8], while H4K20me2 is generated primarily by SUV420H1/KMT5B (which can also produce H4K20me3) and H4K20me3 by SUV420H2/KMT5C [9], [10], [11]. At the cellular level, knockdown of PR-Set7 is associated with mitotic defects and an inability to undergo chromosome condensation, and its targeted ablation in mice is embryonic lethal [12], [13], [14], [15], [16], [17]. In contrast, mice deficient for *Suv420h2* are viable [11], although mouse embryonic fibroblasts lacking this enzyme exhibit defects in telomere maintenance, which is consistent with the accumulation of H4K20me3 in telomeric heterochromatin [18]. Lastly, *Suv420h1* knockout mice die perinatally and show overall growth retardation [11], indicating that the three histone H4-K20 methyltransferases (KMTs) have distinct functions *in vivo*.

Together with KMT loss-of-function studies, biochemical, cell biological, and genetic analyses also suggest that the three methyl states of histone H4K20 confer distinct functions (reviewed in [19]). For instance, they are characterized by non-overlapping distributions within interphase and mitotic nuclei, as well as along chromatin fibers, and exhibit differences during the cell cycle [9], [20]. Specifically, H4K20me1 undergoes a modest elevation during S phase, but is markedly increased as cell progress from G2 through mitosis. This is contrasted by H4K20me2, which is lowest in G2 and achieves its maximum in G1 where it accounts for >90% of K20-methylated histone H4. The least abundant derivative, H4K20me3, undergoes only modest changes during the cell cycle [9]. Nevertheless, the trimethyl derivative of histone H4K20 exhibits marked changes during cell differentiation and ageing [21], [22]. Specifically, analyses in mid-gestation mouse embryos reveal a marked elevation of H4K20me3 in muscle and neural lineages and a corresponding decrease in H4K20me1 as cells became post-mitotic [21]. These findings suggest that the balance of histone H4K20 methylation states is important in the transition from cell proliferation to differentiation, which is further supported by the decrease in H4K20me3 that occurs in malignancy [23], [24], [25].

To address the potential role of histone H4K20 methylation in cellular differentiation, we used a gain-of-function strategy to characterize the SUV420H proteins during myogenesis. At the same time, this provided an opportunity to assess potential differences in the activity of SUV420H1 and SUV420H2, as well as associated splice forms. In particular, SUV420H1 comprises two forms, designated SUV420H1_i1 and SUV420H1_i2, where the latter corresponds approximately to the amino-terminal half of the former and has not been previously characterized. We found that SUV420H proteins differed in their activity at the cellular level and that manipulating histone H4K20 methylation had marked effects on cell differentiation.

## Methods

### Plasmids and RT-PCR

Full-length human *SUV420H1_v1*, *SUV420H1_v2* and *SUV420H2* cDNAs (Mammalian Gene Collection; purchased from Open Biosystems) were subcloned into pEGFP-C1 (BD BioScience), pEGFP-N1 (BD BioScience), and pCI (Promega) that had been modified to encode an amino-terminal hemagglutinin-A epitope tag. In each case, open reading frames were amplified by PCR and their integrity was confirmed by sequencing. A similar approach was used to produce GPF-HP1α (pEGFP-C1) and DsRed2-HP1α (pDsRed2-C1; BD Bioscience) expression plasmids. Gene and protein annotations are based on the Human Genome Nomenclature Committee guidelines (http://www.genenames.org/guidelines.html).

### Cell Culture and Transfection

Mouse C2C12 myoblast cells and C3H 10T1/2 fibroblasts were acquired from the American Type Culture Collection and cultured in DMEM supplemented with 4 mM L-glutamine and either 10% (10T1/2) or 15% FBS (C2C12), which for the latter was designated as growth media (GM). All cells were cultured at 37°C with 5% CO_2_ and transfected at 50%–80% confluency. To characterize SUV420H localization and affects on histone H4K20 methylation, cells were transiently transfected with expression plasmids using PEI transfection reagent (Polysciences, Inc.). For immunofluorescence, cells were seeded on glass cover slips in 6-well plates at a density of ∼1.0×10^6^ cells/ml. After 24 hrs, cells were transfected using an empirically optimized PEI:DNA ratio of 5∶1. For C2C12 cells, 200 µl of serum-free DMEM was added to 1 µg of plasmid DNA, at which point 5 µl of PEI transfection reagent was added and the contents were mixed by ‘vortexing’ for 10s. The same procedure was used for C3H 10T1/2 cells except that 67 µl of serum-free DMEM was added to 1 µg of plasmid DNA. The PEI:DNA mixture was incubated at room temperature for 15 min and then added drop-wise to cover slips that had been washed twice with 2 ml of d-PBS. Cells were processed for immunofluorescence after 24 hrs. For co-transfection of 10T1/2 cells with SUV420H and HP1α, 1 µg of each expression plasmid was used. For differentiation experiments, transfections were carried out using Fugene reagent (Roche). Cells were plated onto ethanol-sterilized glass cover slips so that they were 50 to 80%-confluent the following day and then transfected with 1 µg of specified plasmid construct at a 6∶1 Fugene:DNA ratio according to the manufacturer's instructions. To induce differentiation of C2C12 cells, GM was replaced with DMEM containing 2% horse serum (designated as differentiation media; DM). For time course experiments, cells were transferred to DM 24 hrs post-transfection (assigned as day 0) and then analyzed at 24 hr intervals from days 1 to 5 with media changes every 2 days.

### Immunofluorescence and epifluorescence microscopy

Cells on cover slips were washed with phosphate-buffered saline (PBS) and fixed with freshly prepared 4% paraformaldehyde for 5 min at room temperature, followed by three 5 min PBS washes. For cells transfected with GFP expression plasmids, cover slips were mounted onto glass slides with 20 µl of Mowoil containing 1 µg/ml Hoechst 33258 (Sigma); for immunofluorescence, cells were first permeabilized with 1% TritonX-100 in PBS for 10 min at room temperature. After three 5 min washes in PBS, cells were blocked using 5% BSA in PBS for 1 hr at 4°C, rinsed 3 times with PBS containing 1% BSA, and then incubated with primary antibody (rabbit α-H4K20me3 (AbCam), 1∶100; rabbit α-H4K20me2 (AbCam), 1∶200; rabbit α-H4K20me1 (AbCam), 1∶250; mouse α-HA (Covance), 1∶1000; rabbit α-HA (AbCam), 1∶200; and mouse α-F5D (Developmental Studies Hybridoma Bank), 1∶100) for 1 hr at room temperature. Cells then underwent three 5 min washes with PBS containing 1% BSA prior to incubation with fluorescent secondary antibody (α-mouse Alexa594, 1∶400; α-mouse Alexa488, 1∶400; α-rabbit Alexa594, 1∶400 (Molecular Probes)) and incubated at room temperature for 1 hr in the dark. Last, cover slips were washed three times for 5 min with PBS and then mounted as above. For differentiation experiments, cells were fixed, permeabilized, and immunostained using the α-F5D antibody together with αHA antibody, or no antibody if using GFP-fusion proteins.

### Image collection and analysis

To quantify Myogenin and SUV420H co-expression during C2C12 differentiation, cells were visualized using a Leica DMRE fluorescence microscope. Images were captured with a monochrome 10-bit CCD camera (Qimaging) and collected with Northern Eclipse v6.0 software (Empix Imaging Inc.). Cells were manually counted and data was exported to Excel (Microsoft) where quantitative analyses were performed. High resolution images were acquired on a Zeiss Axioplan 2 microscope equipped with a 12-bit CoolSnap HQ cooled CCD camera (Roper Scientific Inc.). Images were captured using Metamorph 7.5 (Molecular Devices) and deconvolved using Huygens Essentials Deconvolution software (Scientific Volume Imaging). Image reconstruction was performed using Imaris (Bitplane AG).

### Protein extracts and western blotting

For the preparation of protein extracts, cells were recovered via centrifugation (1 min at 13,000 rpm) at room temperature and resuspended in 250 µl of cold lysis buffer containing 50 µl of proteinase inhibitor cocktail (Mammalian PIC, Sigma) and 10 µl of 100 mM phenylmethylsulphonyl fluoride). After 30 min incubation on ice, lysis was completed by sonication (two 10s pulses) and cellular debris was removed by centrifugation at 13,000 rpm for 10 min at 4°C. To prepare acid extracted histones, 10T1/2 cells were detached from 15 cm tissue culture plates by treatment with PBS containing 0.53mmol/l EDTA (10 min at 37°C). Cells were recovered by centrifugation at 1,500 rpm for 5 min (4°C) and washed three times with cold PBS prior to lysis in cold histone acid extraction buffer (1 min at room temperature). Nuclei were harvested by centrifugation at 3,000×g for 1 min, resuspended in 570 µl sterilized water containing 6.4 µl of 4N H_2_SO_4_, and left on ice for 30 min. Nuclear debris was separated from acid-soluble nuclear proteins by centrifugation. The supernatant was transferred to 60 µl of Tris-HCl buffer (pH 8.0) containing 40 µl of 10N NaOH and mixed by vortexing. Protein separation was achieved by SDS-PAGE using either 15% (histone extracts) or 10% (whole cell extracts) separating gels. Protein concentration was determined by Bradford assay and equivalent quantities of protein were resolved and transferred to PVDF membranes, which were then blocked (2 hrs at room temperature) in PBS containing either 5% nonfat milk, 10% nonfat milk, or 5% BSA depending on the antibody. Primary antibody dilutions were as follows: α-H4K20me1, 1∶500 in 5% BSA (AbCam); α-H4K20me2, 1∶1000 in 10% milk (Upstate), α-H4K20me3, 1∶750 in 5% BSA (AbCam); and mouse α-HA, 1∶2000 in 5% milk (Covance). Following an overnight incubation at 4°C, blots were washed 4 times (10 min) in TBST and incubated with secondary antibody (1 hr at room temperature): α-rabbit HRP (1∶5000) or α-mouse HRP (1∶5000 (Cedarlane)). Blots were washed 4 times with Tris-Buffered-Saline-Tween (TBST) for 10 min and then underwent a final rinse with TBS lacking Tween-20. Chemiluminescence was performed as per the manufacturer's instructions (Amersham Biosciences) using Kodak Biomax film.

## Results

### The *SUV420H1* gene encodes a second highly conserved isoform

**Figure 1:**
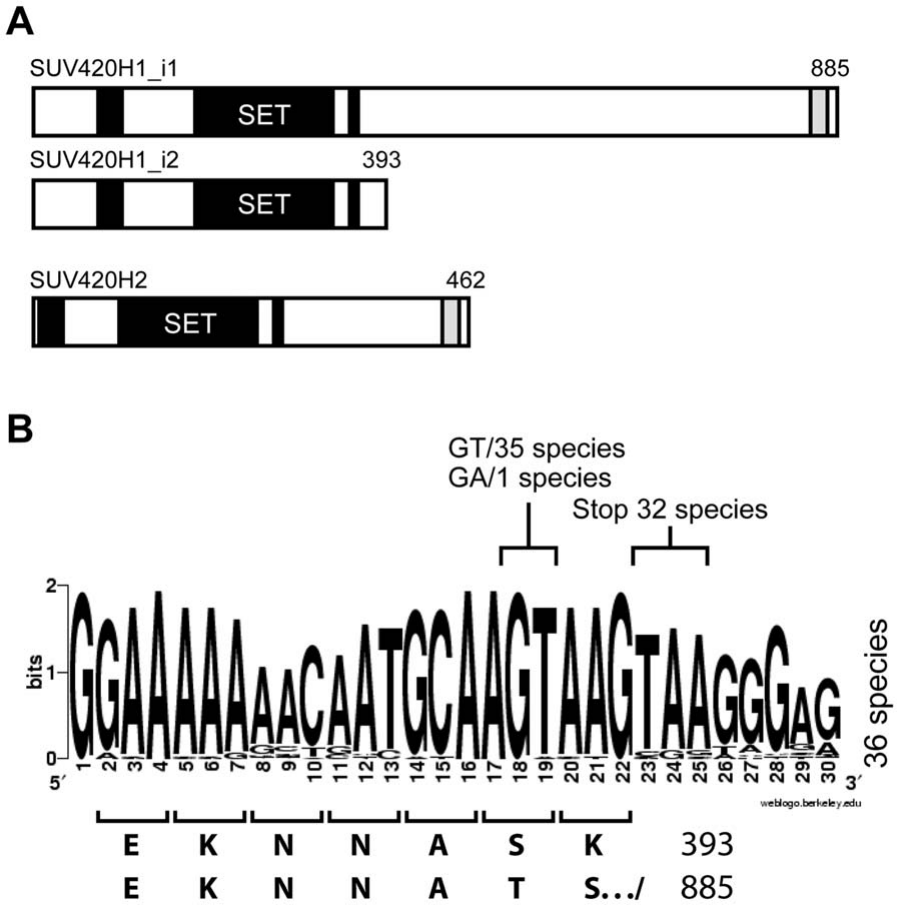
Conservation of a second SUV420H1 isoform. A) Schematic overview of the SUV420H1 isoforms (SUV420H1_i1 and SUV420H1_i2) and SUV420H2. All three SUV420H proteins contain the catalytic SET domain, as well as 2 additional regions of conservation (*black boxes*). The shorter SUV420H1_i2 isoform lacks a 492 amino portion of SUV420H1_i1, which includes a short region of homology with SUV420H2 (*gray box*). B) Homology of the exon 9-intron 9 splice junction is depicted with a WebLogo pictogram (weblogo.berkeley.edu) using sequences aligned from 36 metazoan species (see Figure S1). As a result of alternative splicing, the SUV420H1_i2 protein is identical to SUV420H1_i1 over the first 391 amino acids and encodes only two additional residues (‘S-K’), which are encoded by intron 9 and differ between the two isoforms.

Genetic screens for novel regulators of position-effect-variegation (PEV) in *Drosophila* led to the discovery of *suv420*, a member of the SET-domain family of lysine methyltransferases (KMTs), and facilitated the identification of two mammalian homologues, SUV420H1 and SUV420H2 [10]. Although genetic and biochemical data support the ability of these KMTs to trimethylate histone H4-lysine 20 (H4K20me3) *in vitro* and in transfected cells [10], [26], SUV420H1 appears largely responsible for the catalysis of H4K20me2 *in vivo*, while SUV420H2 activity accounts for the production of H4K20me3 [11], [26]. In the current study, we have examined how differences in the primary structure of these KMTs influence their biological activity. Importantly, we have included a second SUV420H1 isoform that is annotated in NCBI Build36 of the human genome. This protein (referred to as SUV420H1_i2) is identical to the canonical form (designated SUV420H1_i1) over the first 391 residues and arises by retention of an intron that creates an alternatively spliced mRNA encoding only two additional amino acids (Figure 1A). As a result of this truncation, SUV420H1_i2 contains the catalytic SET domain and two additional regions of homology (Figure 1A, *filled boxes*), but lacks a third segment that is present in both SUV420H1_i1 and SUV420H2 (Figure 1A, *gray box*).

As a first step in evaluating the authenticity of the SUV420H1_i2 isoform, we sought to establish its evolutionary conservation. To this end, multiple genome databases (HTGS, GSS, WGS; 36 species in total) were queried and sequence alignments were carried out using the region that spans the exon 9-intron 9 junction (Figure 1B and Figure S1A). This revealed perfect conservation of the splice donor with only one occurrence of an atypical GA splice site (*Dasypus novemcinctus*). Similarly, conservation of the stop codon is also clearly evident, being present in 32 of 36 sequences (Figure 1B and Figure S1A). The only exceptions were *Gallus gallus, Anolis carolinensis*, *Monodelphis domestica,* and *Xenopus tropicalis*, which retain the splice site but encode an additional 4, 16, 17, or 20 amino acids before encountering a stop codon. Importantly, analysis of the Expressed Sequence Tag (EST) database indicated that the *SUV420H1_v2* mRNA is generated *in vivo* and was detected in multiple tissues from diverse species (data for mouse and human is shown in Figure S1C). Lastly, homology included the 3′-UTR, where there was a region of conservation downstream of exons 9 (3′-UTR for *SUV420H1_v2*) and 10 (3′-UTR for *SUV420H1_v1*) that was not observed flanking other exons of the *SUV420H1* gene (Figure S1B). Based on the widespread conservation of the shorter SUV420H1_i2 protein, this isoform was used in comparative analyses together with SUV420H1_i1 and SUV420H2 to assess their localization and activity *in situ*.

### The SUV420H1_i2 isoform exhibits a localization that is distinct from SUV420H1_i1 and SUV420H2

**Figure 2:**
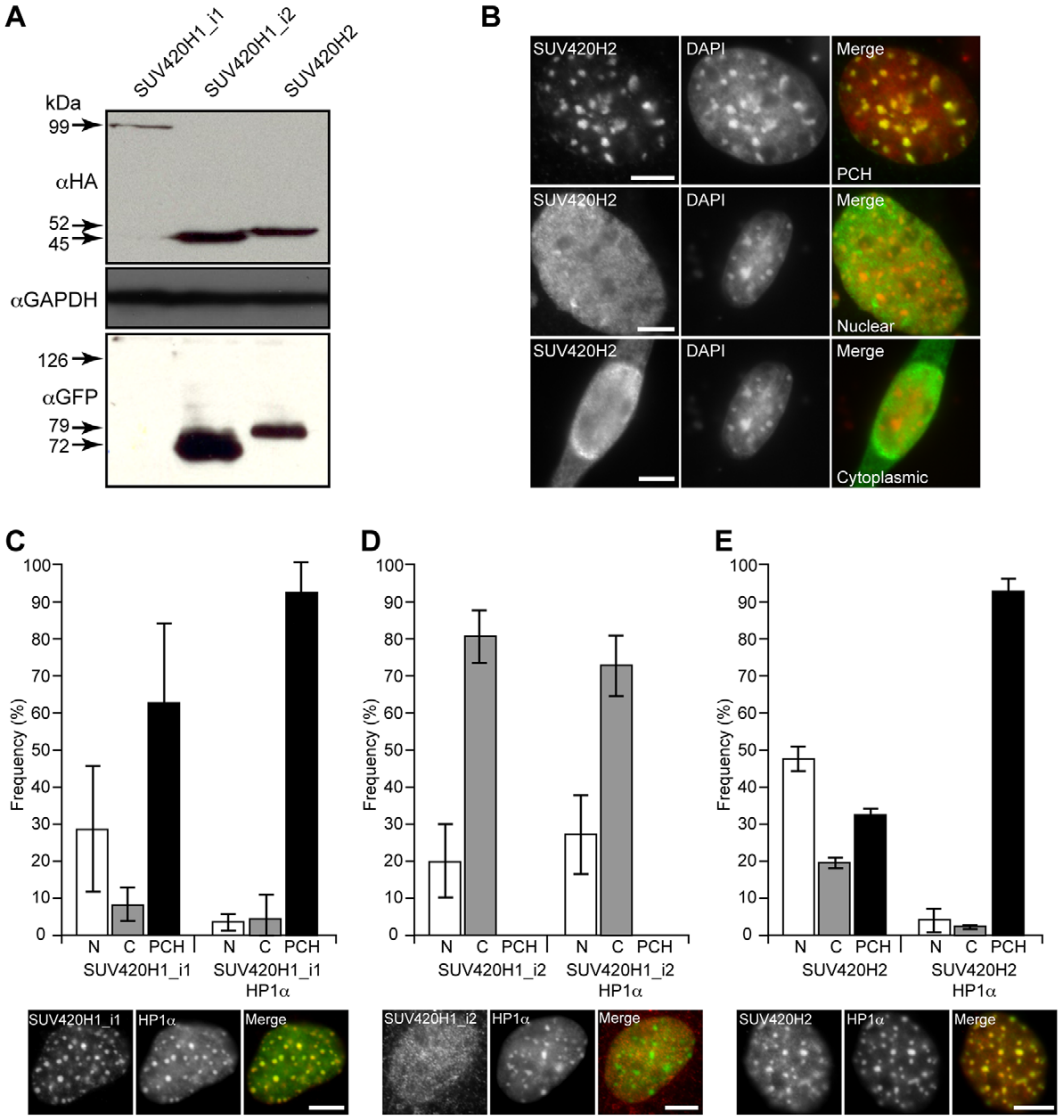
Expression and localization of recombinant SUV420H proteins. A) C3H 10T1/2 cells were transfected with expression plasmids encoding SUV420H1_i1, SUV420H1_i2, and SUV420H2 with an N-terminal HA-tag or with GFP fused to either the N- or C-terminus. Representative western blots are shown for HA-tagged (*upper panel*) and GFP versions of these KMTs (*lower panel*). SDS-PAGE was carried out with equivalent amounts of total cellular protein (a GAPDH immunoblot is included as a loading control). With the exception of SUV420H1_i1-GFP, which could not be detected, all recombinant proteins were of the expected size. B) Representative micrographs depict cytoplasmic, diffuse nuclear, or pericentric heterochromatin localization for HA-SUV420H2. Gray scale images are shown for individual channels and the merged image depicts SUV420H2 (*green*) and DAPI (*red*). C-E) Each SUV420H protein was scored based on its localization (as in B) and the resulting data is shown in a bar graph (N, nuclear; C, cytoplasm; and PCH, pericentromeric heterochromatin) in the presence or absence of HP1α co-transfection. Error bars represent standard error of the mean from two independent experiments. The micrographs depict representative images of HA-SUV420H and GFP-HP1α co-expression. Gray scale images are shown for individual channels and the merged image depicts SUV420H2 (*red*) and HP1α (*green*). Controls involving GFP/HP1α-dsRED2 or dsRED2/HP1α-GFP established that the fluorescent protein moieties did not influence localization.

Initial description of the SUV420H proteins focused on SUV420H1_i1 and SUV420H2, and found that both localize to regions of pericentromeric heterochromatin (PCH) [10]. In each case, deletion analyses implicated the carboxy-terminus, which is absent from SUV420H1_i2, in conferring this distribution [10]. To evaluate the impact of these differences in primary structure on cellular distribution, we employed immunofluorescence to compare the localization of both SUV420H1 isoforms and SUV420H2. For this purpose, derivates of each KMT were made that contained an amino-terminal HA epitope tag (Figure 2A, *upper*), as well as versions containing GFP fused to the amino or carboxy-terminus (Figure 2A, *lower*), and sizes were verified by western blotting. In these experiments, SUV420H1_i1 was consistently expressed at a lower level than either SUV420H1_i2 or SUV420H2, but could be detected by immunofluorescence (addressed below) where it displayed a 2 to 3-fold lower transfection efficiency. Upon examining the cellular distribution of each KMT, they exhibited multiple localization patterns that included PCH, diffuse nuclear staining, or simultaneous presence in both the nucleus and the cytoplasm (Figure 2B).

The current model for localization of SUV420H proteins involves their recruitment to constitutive heterochromatin through a direct interaction with HP1α via its binding to H3K9me3 [10]. Accordingly, cells lacking H3K9me3 in constitutive heterochromatin owing to a knockout of the underlying KMTs, *Suv39h1* and *Suv39h2*, also lack H4K20me3 in these domains [10]. The distribution of each SUV420H protein was therefore quantified in the absence or presence of HP1α co-expression (Figure 2C-2E). As expected, exogenous SUV420H1_i1 (Figure 2C) and SUV420H2 (Figure 2E) displayed PCH distribution, but were also characterized by a fraction of cells with a diffuse nuclear pattern and a smaller percentage of cells that exhibited cytoplasmic staining. These latter fractions were consistently greater for SUV420H2 and may relate to its higher expression level (Figure 2A). In contrast, SUV420H1_i2 only exhibited diffuse cytoplasmic or nuclear distribution (Figure 2D). Interestingly, co-expression of HP1α caused the redistribution of SUV420H1_i1 (Figure 2C) and SUV420H2 (Figure 2E) such that nearly all (>90%) co-transfected cells displayed localization to PCH. As a result, the cytoplasmic or diffuse nuclear distributions likely occur when levels of SUV420H1_i1 and SUV420H2 exceed the endogenous capacity of HP1 proteins to direct PCH localization. The distribution of SUV420H1_i2, however, was largely unaffected by HP1α expression, although there was a trend towards an increased ‘nuclear diffuse’ fraction (Figure 2D). Together, these observations indicate that while HP1α is rate limiting for localization of SUV420H1_i1 and SUV420H2 to constitutive heterochromatin, SUV420H1_i2 is under distinct control and lacks determinants for PCH localization.

### SUV420H1_i2 differs from SUV420H1_i1 and SUV420H2 in where it catalyzes H4K20me3

**Figure 3:**
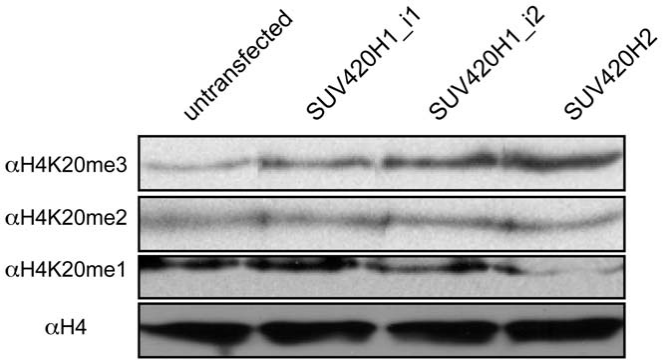
Western analysis of histone H4K20 methyl derivatives following expression of exogenous SUV420H proteins. C3H 10T1/2 cells were transfected with HA-tagged expression plasmids for SUV420H1_i1, SUV420H1_i2, and SUV420H2, and used for the preparation of acid extracted histones. Equivalent amounts of histone protein from *SUV420H*-transfected and untransfected cells were used in western blotting with antibodies specific for H4K20me1, H4K20me2, H4K20me3, or total histone H4. Expression of each SUV420H KMT led to an increase in H4K20me3 levels that was proportional to the transfection efficiency, a corresponding decrease in H4K20me1 for SUV420H1_i1 and SUV420H2, and no affect on H4K20me2. It should be noted that the H4K20me2 modifications accounts >80% of histone H4 methylated species in asynchronous cells [9], making it difficult to detect modest changes in levels.

**Figure 4:**
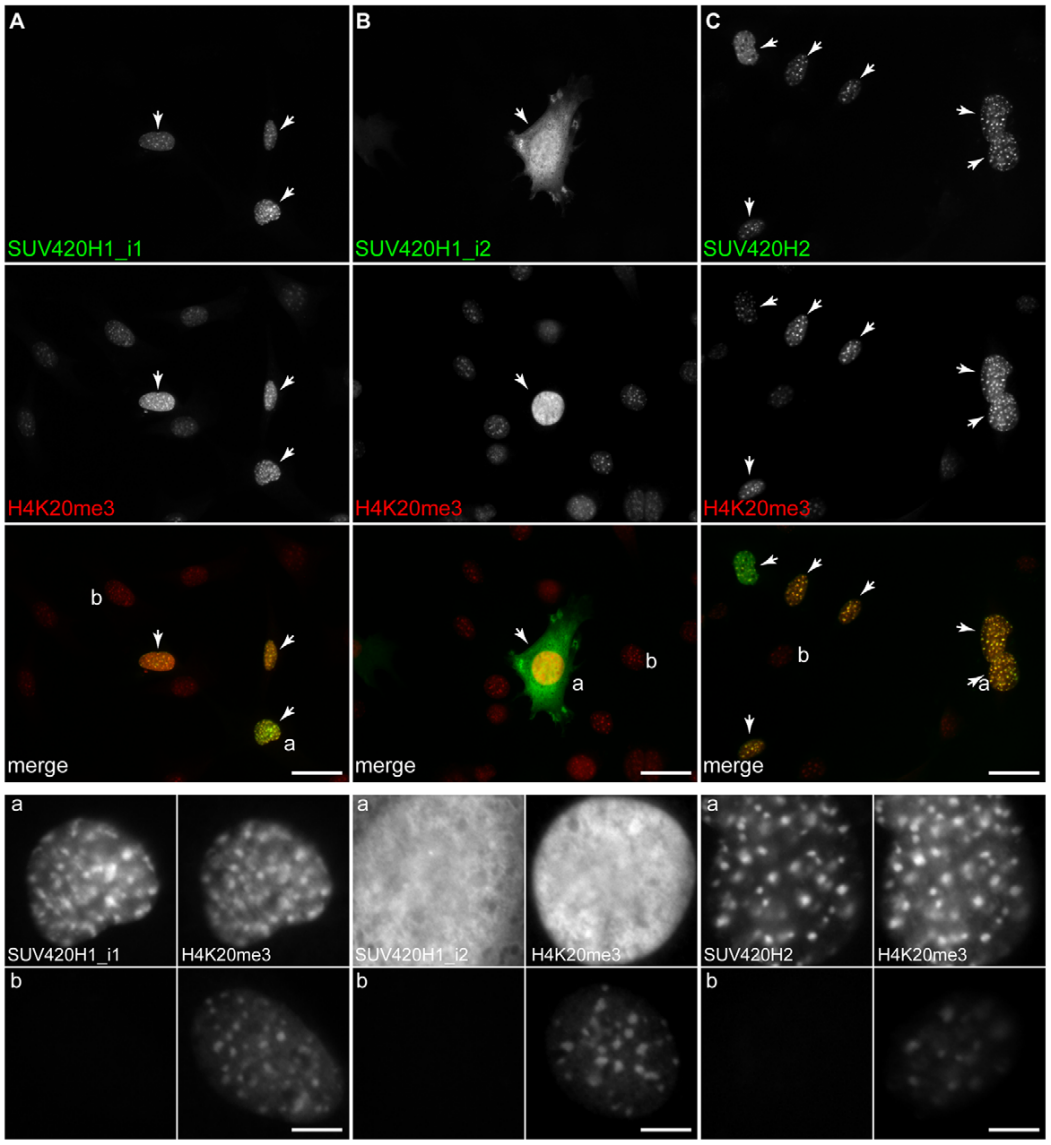
Affect of exogenous SUV420H expression on H4K20me3. C3H 10T1/2 cells were transfected with expression plasmids encoding SUV420H1_i1, SUV420H1_i2, and SUV420H2 with an N-terminal HA-tag or with GFP fused to either the N- or C-terminus and processed for immunofluorescence using antibodies specific for H4K20me3. Representative micrographs are shown for N-terminal GFP fusions of (A) SUV420H1_i1, (B) SUV420H1_i2, and (C) SUV420H2. In each panel, 40× field shots are shown that display grayscale images for SUV420H and H4K20me3, and the corresponding merged image (SUV420H, *green*; H4K20me3, *red*). The lower panels depict representative transfected (*a*) or non-transfected (*b*) cells that have been copied from the 40× micrograph and include grayscale images for the recombinant enzyme and H4K20me3. Scale bars are 50 µm (*upper panels*) or 10 µm (*lower panels*).

**Figure 5:**
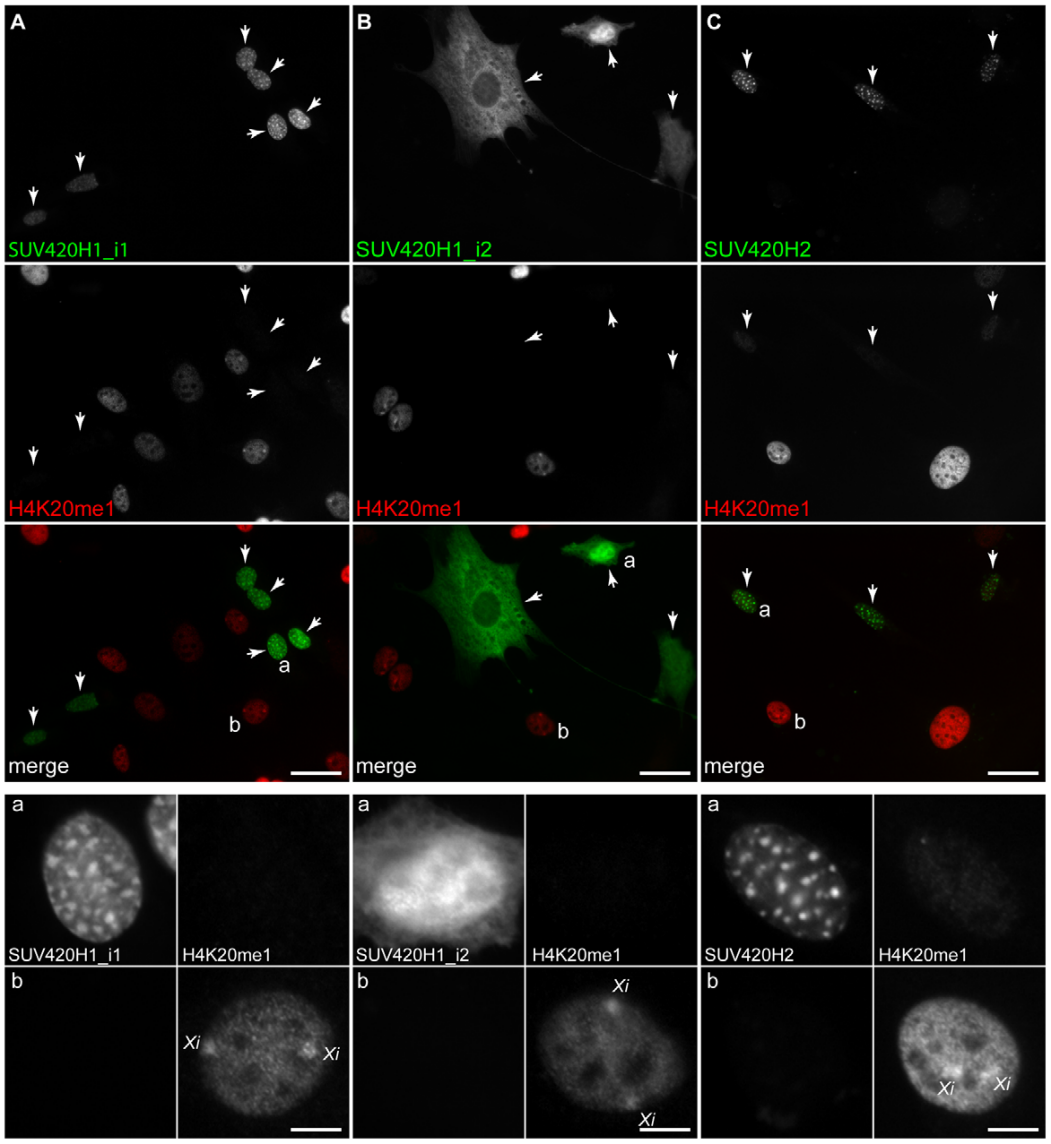
Affect of exogenous SUV420H expression on H4K20me1. C3H 10T1/2 cells were transfected with expression plasmids encoding SUV420H1_i1, SUV420H1_i2, and SUV420H2 with an N-terminal HA-tag or with GFP fused to either the N- or C-terminus and processed for immunofluorescence using antibodies specific for H4K20me1. Representative micrographs are shown for N-terminal GFP fusions of (A) SUV420H1_i1, (B) SUV420H1_i2, and (C) SUV420H2. In each panel, 40× field shots are shown that display grayscale images for SUV420H and H4K20me1, and the corresponding merged image (SUV420H, *green*; H4K20me1, *red*). The lower panels depict representative transfected (*a*) or non-transfected (*b*) cells that have been copied from the 40× micrograph and include grayscale images for the recombinant enzyme and H4K20me1. Enrichment of H4K20me1 on the inactive X chromosome (*Xi*) is indicated. Scale bars are 50 µm (*upper panels*) or 10 µm (*lower panels*).

Given the marked differences in localization of SUV420H1_i2 in comparison to SUV420H1_i1 and SUV420H2, we sought to determine if these influence the nuclear distribution of their methylated products. As a first step, we assessed the affect of exogenous SUV420H expression on global levels of histone H4-K20 methyl derivatives by western blotting (Figure 3), which indicated each enzyme could induce an increase in H4K20me3 levels that was proportional to their relative transfection efficiency (SUV420H2>SUV420H1_i2≫SUV420H1_i1). For both SUV420H1_i2 and SUV420H2, we also observed a corresponding decrease in H4K20me1 (Figure 3). Although this reduction was not apparent with SUV420H1_i1, likely because of its lower overall expression level (Figure 2A), its activity was clearly apparent in immunofluorescence experiments (addressed below). As we did not observe differences in H4K20me2 levels upon SUV420H expression (addressed in discussion), co-immunofluorescence studies focused on analyses of H4K20me3 and H4K20me1. Exogenous expression of SUV420H1_i1 (Figure 4A), SUV420H1_i2 (Figure 4B), and SUV420H2 (Figure 4C) in C3H 10T1/2 mouse fibroblast cells resulted in increased H4K20me3, regardless of localization pattern. For SUV420H1_i1 and SUV420H2, transfected cells retained their characteristic pattern of enrichment at PCH, but also displayed more widespread H4K20me3 elevation. Consistent with its diffuse nuclear localization, SUV420H1_i2 caused a marked increase in H4K20me3 throughout the nucleus with no obvious elevation in regions of constitutive heterochromatin (Figure 4B). This further distinguished the activity of this KMT from SUV420H1_i1 and SUV420H2, both of which caused an increase in H4K20me3 in regions of PCH (Figure 4A and C, compare *a* and *b* panels). As expected, H3K9me3 levels were unaltered in cells expressing any of the SUV420H enzymes (data not shown). Lastly, we observed that expression of all isoforms of SUV420H resulted in an obvious decrease in H4K20me1 throughout the nucleus, further supporting the notion that H4K20me1 is the substrate for catalysis of H4K20me3 by all three SUV420H proteins (Figure 5A-C) [9], [11]. In addition, the fact that the conversion of H4K20me1 to H4K20me3 occurs throughout the nucleus indicates that the activity of SUV420H1_i1 (Figure 5A, *lower* panels) and SUV420H2 (Figure 5C, *lower* panels) is not necessarily limited to constitutive heterochromatin. Consistent with this, the enrichment of H4K20me1 that occurs in facultative heterochromatin on the inactive X chromosome (Figure 5A-C, labeled *Xi* in *lower* panels) was also lost upon exogenous SUV420H expression. Expression of GFP alone had no affect on levels of H4K20me1 or H4K20me3 (Figure S2). These findings therefore indicate SUV420H1_i2 differs in where it catalyzes H4K20me3 formation, providing another parameter that is distinct from SUV420H1_i1 and SUV420H2.

### SUV420H proteins have distinct affects on the differentiation of C2C12 cells

**Figure 6:**
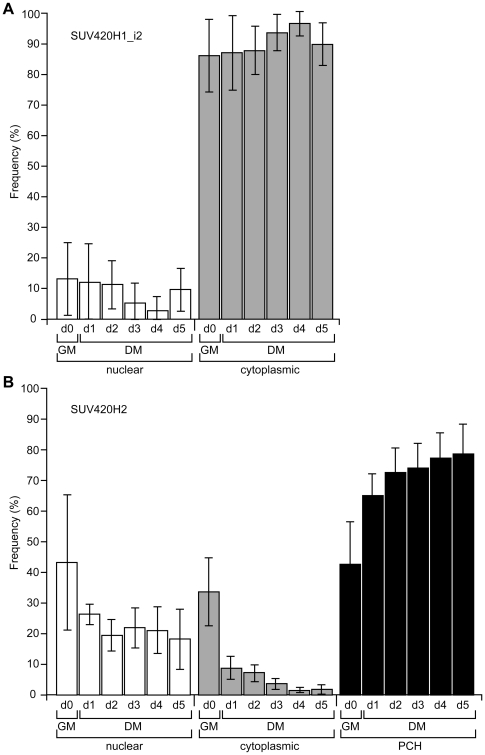
Localization of exogenous SUV420H proteins during myogenesis in C2C12 cells. The C2C12 myoblast cell line was transfected with expression plasmids for SUV420H1_i2 (A) and SUV420H2 (B). The localization of recombinant proteins was monitored by immunofluorescence at day 0 (d0; 24 hr post-transfection) in GM and over a 5 day (d1-d5) differentiation time course in DM. Graphs provide an overview of SUV420H localization (C, cytoplasmic, *gray bars*; N, nuclear, *open bars*; or PCH, pericentromeric heterochromatin, *closed bars*). Data is plotted as a percentage (*Frequency*) of total SUV420H-expressing cells displaying the indicated distribution from day 0 to day 5. Error bars represent standard deviation from three independent experiments and significant differences are noted in the text. As noted in the text, we were unable to express SUV420H1_i1 in the C2C12 cell line. The compiled data is based on the analysis of 56,939 (SUV420H1_i2) and 64,464 cells (SUV420H2).

We previously described an inverse relationship between H4K20me1 and H4K20me3 in the myotome of mid-gestation mouse embryos, which was recapitulated in primary myoblast cultures. Specifically, H4K20me1 levels declined and those for H4K20me3 increased during myogenic differentiation [21]. These changes in histone H4-K20 methylation were also observed in the C2C12 myogenic model system [21], which can be induced to differentiate by switching cell cultures from regular growth media (GM) to differentiation media (DM) containing 2% serum (see *Experimental Procedures*). We therefore wanted to determine if facilitating this transition in histone H4K20 methylation (see Figures 4 and 5) had an impact on C2C12 cell differentiation. To this end, C2C12 cells were transfected with SUV420H expression plasmids and then assessed for the differentiation marker Myogenin (Myog) by co-immunofluorescence at day 0 while still in GM (24 hrs post transfection) or at days 1 to 5 after transfer to DM. The percentage of Myog-positive cells was determined from the population of non-transfected cells and cells expressing the SUV420H protein. At the same time, three other parameters were assessed: we determined overall transfection efficiency and retention of transfected cells, as well as monitored the localization of SUV420H proteins during the differentiation time course (Figure 6). Paradoxically, this indicated that neither HA or GFP-tagged SUV420H1_i1 could be efficiently expressed in C2C12 cells, despite being able to detect the recombinant protein in 10T1/2 cells by immunofluorescence (Figures 4 and 5) and western blotting (Figure 2A, *upper panel*). Moreover, this inability was also apparent 4 and 8 hrs post-transfection, suggesting the protein is never made or is rapidly degraded, and it was therefore not included in subsequent analyses. Of the two remaining proteins, SUV420H2 was consistently characterized by higher transfection efficiency than SUV420H1_i2 (20.9% versus 5.5% at day 0) and a greater proportion of SUV420H2-transfected cells were retained over the course of differentiation (5.4% versus 1.5%). This decline occurred from day 0 to day 2, after which the fraction of transfected cells was stable. Together, these differences suggest the exogenous SUV420H1 proteins are either deleterious to the expressing cell or that their levels are tightly regulated.

In terms of localization, SUV420H1_i2 did not change from its predominantly cytoplasmic distribution, which remained significantly higher (*p<0.05*) than the nuclear fraction over the differentiation time course (Figure 6A). On the other hand, although SUV420H2 was similar at day 0 to that described in 10T1/2 cells (Figure 2E), the fraction of cells displaying cytoplasmic or diffuse nuclear distribution declined while those exhibiting localization to pericentric heterochromatin underwent a significant increase (*p<0.05*) from day 0 to day 1, and continued to increase throughout (Figure 6B). Similarly, with the exception of day 0, the heterochromatic portion was significantly (*p<0.05* to *p<0.005*) elevated compared to nuclear and cytoplasmic fractions at all time points.

**Figure 7:**
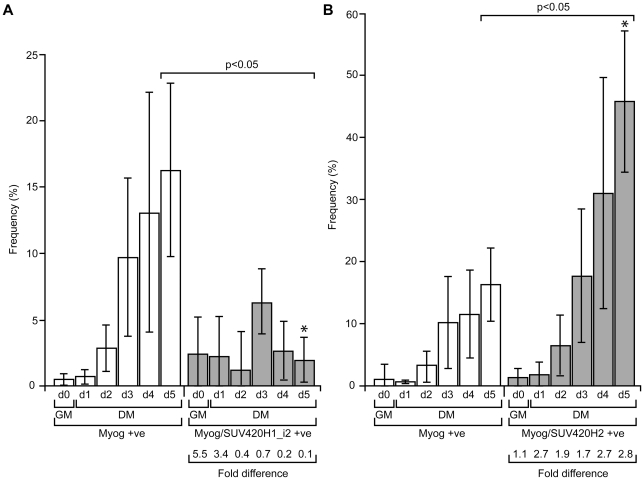
Affect of SUV420H expression on myogenic differentiation in C2C12 cells. The C2C12 myoblast cell line was transfected with expression plasmids for SUV420H1_i2 (A) and SUV420H2 (B). Transfected cells were monitored for expression of endogenous Myogenin (Myog) and exogenous SUV420H proteins by co-immunofluorescence on day 0 (d0; 24 hr post-transfection) in GM and days 1 through 5 (d1-d5) in DM. Graphs indicate the percentage of cells expressing Myog (detected with the F5D monoclonal antibody; *open bars*) and the percentage of SUV420H-expressing cells positive (*+ve*) for Myog (*gray bars*). The values listed below each graph depict the ratio of SUV420H/Myog double positive cells to cells singly positive for Myog at each day. Error bars represent standard deviation from three independent experiments and significant differences are noted between the d5 values for Myog and Myog/SUV420H (*brackets*), and when comparing Myog/SUV420H1_i2 to Myog/SUV420H2 (*asterisks*). The compiled data is based on the analysis of 56,939 (SUV420H1_i2) and 64,464 cells (SUV420H2).

Differences in the activity of SUV420H1_i2 and SUV420H2 continued upon examining their influence on C2C12 myogenesis. We observed a similar percentage of Myog-positive cells over the course of differentiation when considering only the non-transfected cells (<1% at day 0 and ∼16% by day 5; Figure 7A and B). In cells expressing SUV420H1_i2 (Figure 7A), however, there was an increase in Myog-positive cells even prior to the addition of DM (∼5.5-fold), which suggests this enzyme can induce precocious differentiation. Nevertheless, the situation changes in DM where we observed a steady decline in SUV420H1_i2/Myog double positive cells (∼2% at day 5), while SUV420H2 expressing cells maintained a higher fraction (∼46% at day 5) of Myog-positive cells than the bulk culture (compare Figure 7A to 7B). Expressing the data as the fold difference between double-positive cells versus Myog single positives clearly illustrates the effects of SUV420H1_i2 and SUV420H2. Despite inducing precocious appearance of Myog, the ratio of Myog to Myog/SUV420H1_i2-double positive cells steadily declines from 5.5 on day 0 to 0.12 on day 5, representing an approximately 46-fold change. In contrast, SUV420H2 maintained an increased population of double-positive cells at all time points tested (2.2-fold on average). Thus, even though SUV420H1_i2 and SUV420H2 have similar effects on global levels of H4K20me1 and H4K20me3 when expressed exogenously (Figures 4 and 5), they are associated with distinct activities and only the latter supports enhanced differentiation.

## Discussion

In mammalian cells, methylation of lysine 20 on histone H4 is carried out by PR-Set7 (SETD8, SET8, KMT5A), SUV420H1 (KMT5B), and SUV420H2 (KMT5C) (reviewed in [19]). Although the latter two display a high level of identity to each other (∼58% over the catalytic domain) and the *Drosophila* suv420h protein, they are distantly related to the PR-Set7 protein in evolutionary terms. In the current study, we characterized a second SUV420H1 isoform, designated SUV420H1_i2, and found it has markedly different activity when compared to the originally described SUV420H1_i1 and SUV420H2 proteins. From a primary structure standpoint, SUV420H1_i2 represents a truncated version of SUV420H1_i1 and is missing the carboxy terminal region that has been implicated in interaction with HP1α and the RB1 tumor suppressor protein [10], [27], [28]. This includes a ‘PxVxL’ motif (PKVVL at position 480), which has been shown to confer interaction with HP1α in numerous proteins [29]. The SUV420H2 protein contains a related PHCRL sequence at position 364 that is present within a short region of the protein known to confer interaction with HP1α and localization to PCH [10], [27], [28]. Consistent with these features, the shorter SUV420H1_i2 isoform did not localize to constitutive heterochromatin and its distribution was unaffected by HP1α co-expression, suggesting that it functions in a distinct histone H4-K20 methylation pathway. The SUV420H1_i2 protein can be further distinguished from SUV420H1_i1 by lacking all predicted nuclear localization signals, likely accounting for its presence in the cytoplasm. Moreover, these differences in carboxy-termini had a marked effect on the expression of the two isoforms—SUV420H1_i1 was expressed at lower levels in 10T1/2 cells and exogenous protein could not be efficiently detected at any time following transfection of the C2C12 cell line. As a result, the carboxy-terminal segment of SUV420H1_i1, or the mRNA sequence encoding it, may be subject to post-translational or post-transcriptional control. In addition, the SUV420H1_i2 protein could not achieve the same transfection efficiency as SUV420H2 (in either 10T1/2 or C2C12), together indicating that exogenous activity of the SUV420H1 proteins is less tolerated, and may therefore affect cell cycle progression or survival.

Expression of individual SUV420H proteins led to increased levels of H4K20me3 and a corresponding decrease in H4K20me1 when assessed by either immunofluorescence or western blotting. This is consistent with PR-Set7 knockdown and gene-targeting experiments that indicate H4K20me1 is a substrate for progressive di- and trimethylation by the SUV420H proteins [9], [11], [17], [26]. However, ablation of the *Suv420h1* gene leads to a specific decrease in H4K20me2 (and an increase in H4K20me1), indicating that SUV420H1 acts primarily as a dimethylase *in vivo* [11]. This disparity is not unexpected for two reasons. First, both SUV420H1 and SUV420H2 are capable of catalyzing H4K20me3 and can also catalyze H4K20me3 *in vitro* from an unmethylated substrate [10], [26]. This flexibility in product specificity is similar to that of *Toxoplasma gondii* PR-Set7 (a.k.a. SETD8) [30] and *S. pombe* set9p, which can catalyze all three histone H4-K20 methyl derivatives [31], and with the finding that human PR-Set7 can be converted to a dimethylase with a single amino acid substitution [32]. Second, in dividing cells where the G_1_ population predominates, H4K20me2 already accounts for ∼90% of histone H4-K20 methylated species [9]. As a result of its overall abundance, it would be difficult to affect a substantive increase in H4K20me2 levels and it therefore remains possible that exogenous SUV420H1 expression may catalyze this modification on a limited scale.

Expression of SUV420H1_i2 and SUV420H2 were associated with distinct outcomes during myogenesis, despite both causing elevated H4K20me3 and reduced H4K20me1. Nevertheless, the two enzymes differed significantly in where they directed H4K20me3. The widespread catalysis of H4K20me3 by SUV420H1_i2 was associated with precocious appearance of Myog in C2C12 cells, which is an early marker for myogenic differentiation [33]. In this model system, differentiation is initiated from the G_1_ phase of the cell cycle upon serum withdrawal [34], [35], which would coincide with peaks in H4K20me2 and H4K20me3, and declining levels of H4K20me1 [9]. This acute change in histone H4-K20 methylation differs, however, from the opposing trend in H4K20me1 and H4K20me3 levels observed during C12C12 differentiation, which occurs gradually over several days in DM [21]. As a result, the precocious expression of Myog induced by exogenous SUV420H1_i2 (and to a lesser extent by SUV420H2) may derive from its suppression of H4K20me1 levels. In dividing cells, this modification normally increases steadily through S-phase before undergoing a marked elevation during mitosis [9], and its catalysis by PR-Set7 is required for progression through S-phase and G_2_/M [12], [13], [14], [15], [16], [17]. Consistent with this idea, PR-Set7 knockdown and the resulting decrease in levels of H4K20me1 caused spontaneous differentiation of K562 myelogenous leukemia cells [36]. Collectively, these data suggest that cell cycle-associated changes in histone H4-K20 methylation play an important role in exit from the cell cycle and initiation of differentiation.

In contrast to the acute effect of SUV420H1_i2 expression, SUV420H2 enriched for Myog-positive cells over the entire course of differentiation (2.8-fold higher by day 5). This was accompanied by increased localization of this enzyme to PCH, which correlates with the gradual increase in H4K20me3 observed in these regions during myogenesis *in vivo* and *in situ* [21]. From a mechanistic standpoint, these observations indicate SUV420H2 promotes the myogenic program through the catalysis of H4K20me3 in regions of PCH. This behavior is clearly different from the inhibition of C2C12 differentiation that occurs upon expression of SUV39H1 [37] and EZH2 [38]. Moreover, transgenic mice over-expressing *Suv39h1* display impaired erythroid differentiation both *in vivo* and when cells are cultured *ex vivo* where they become immortalized [39], and EHZ2 has an essential role in maintaining the pluripotent/undifferentiated state of embryonic stem cells [40], [41], [42]. This suggests their methylated products, H3K9me3 for SUV39H1 [43], [44] and H3K27me2/3 for EZH2 [45], [46], [47], [48], [49], function differently than SUV420H and H4K20me3 in regulating differentiation. Unlike H3K9me3 and H3K27me3, which have been associated with repression of specific loci on a genome wide scale, H4K20me3 showed no obvious association with active or repressed promoters [50]. Given the ability of H4K20me3 to induce compaction of *in vitro* reconstituted chromatin [51], this modification could also serve a unique role in chromatin structure that is not conferred by H3K9me3 or H3K27me3.

The distinct roles for SUV39H and SUV420H proteins are somewhat surprising given localization to PCH by the latter is dependent on H3K9me3 catalyzed by the former [10]. Nevertheless, we did not observe a correlation between H4K20me3 and H3K9me3 levels during differentiation, and the two modifications display distinct dynamics during embryogenesis [21]. Microarray analyses in C2C12 cells also establish that these KMTs display different expression profiles—SUV420H1 and SUV420H2 stay constant or increase, while EZH2 and SUV39H1 decline [52], [53], which has also been independently established for the latter two enzymes [37], [38], [54], [55]. Moreover, exogenous expression of HP1 proteins (α, β, and γ) inhibits myogenic differentiation in the C2C12 model system [56]. It therefore appears that a basal level of H3K9me3 may be required for initial SUV420H recruitment, but the ensuing catalysis of H4K20me3 maintains the differentiated state without a parallel increase in H3K9me3. Lastly, it is noteworthy that H4K20me3 accumulates in ageing tissue of multiple lineages [22] and it will therefore be important to be determine if SUV420H/H4K20me3 function in a similar manner in other models of cell differentiation.

The accumulation of histone H4K20me3 during differentiation and ageing, and the behavior of SUV420H proteins described in the present study are relevant to understanding the role of this modification in cancer. In particular, a number of studies indicate that H4K20me3 levels decline in tumor samples and, more recently, in preneoplastic lesions [23], [24], [25]. Coincident with this change in H4K20me3, levels of SUV420H2 are reduced [24], [25], which draws an important parallel with the affect of this enzyme on differentiation. Specifically, the unique ability of SUV420H2, when compared to SUV420H1, to enhance myogenic differentiation suggests its decrease may contribute to de-differentiation or an inability to maintain the differentiated state. Moreover, given the dynamics of histone H4 K20 methylation and their importance to cell cycle progression [9], [12], [13], [14], [15], [16], [17], any imbalance may contribute to the deregulated cell cycle control that characterizes oncogenic transformation [57], [58]. Taken together with the observation that attenuation of SUV420H activity leads to compromised genomic integrity [11], [18], [27], [59], perturbations in histone H4K20 methylation have the capacity to influence multiple aspects of cancer cell biology.

## Supporting Information

Figure S1 Analysis of evolutionary conservation at the *SUV420H1* locus. A) Multispecies alignment of the exon 9-intron 9 junction. Alignments were carried out using genomic sequences from 36 metazoan species, all of which had the splice donor, with a single occurrence of an atypical ‘GA’ donor site. Only 4 species lacked the conserved stop codon, but each contained an in-frame stop codon that added anywhere from 4-20 amino acid residues. B) Genome alignments were carried out using the Evolutionarily Conserved Regions (ECR) browser of the NCBI DCODE package (http://ecrbrowser.dcode.org) and human *SUV420H1* (chr11:67680083-67737360) as the query sequence. Output from the ECR browser is shown for a region of the human *SUV420H1* gene spanning exons 5 to 10 (exons are depicted in blue, introns in pink, UTRs in yellow, repetitive elements in green, and intergenic regions in red). The sequence 3′ of exon 9, is annotated as a UTR and shows a higher degree of conservation than regions downstream of other exons (including exons 1-4, not shown). Alignments include the corresponding region from the genomes of *Rattus norvegicus*, *Mus musculus*, *Bos taurus*, *Canis familiaris*, and *Rhesus macaques*. C) Expressed sequence tags for the SUV420H1 isoforms. A 30aa segment at the C-terminus of each SUV420H1 isoforms was used to analyze the mouse and human components of the Expressed Sequence Tag (EST) database using tBlastn. The accession numbers and position of the coded sequence within the EST are shown. In each case, the presence of the stop codon was verified and the source tissue or cell line is indicated.
(8.14 MB TIF)

Figure S2 Affect of exogenous GFP expression on H4K20me1 and H4K20me3. C3H 10T1/2 cells were transfected with the pEGFP-N1 expression plasmid (Clontech) and processed for immunofluorescence using antibodies specific for H4K20me1 (A) or H4K20me3 (B). In each case, the upper panel shows a representative micrograph of adjacent cells, where the cell on the left expresses GFP. The lower panels depict staining with H4K20me1 or H4K20me3 antibodies. Enrichment of H4K20me1 on the inactive X chromosome (Xi) is indicated. Scale bar is 10 µm.
(8.38 MB TIF)
