# Supplementary figures and images for: Comparative Analyses of SUV420H1 Isoforms and SUV420H2 Reveal Differences in Their Cellular Localization and Effects on Myogenic Differentiation
Source: PLoS One. 2010 Dec 29;5(12):e14447. doi: 10.1371/journal.pone.0014447 (PMC3012056; doi:10.1371/journal.pone.0014447)

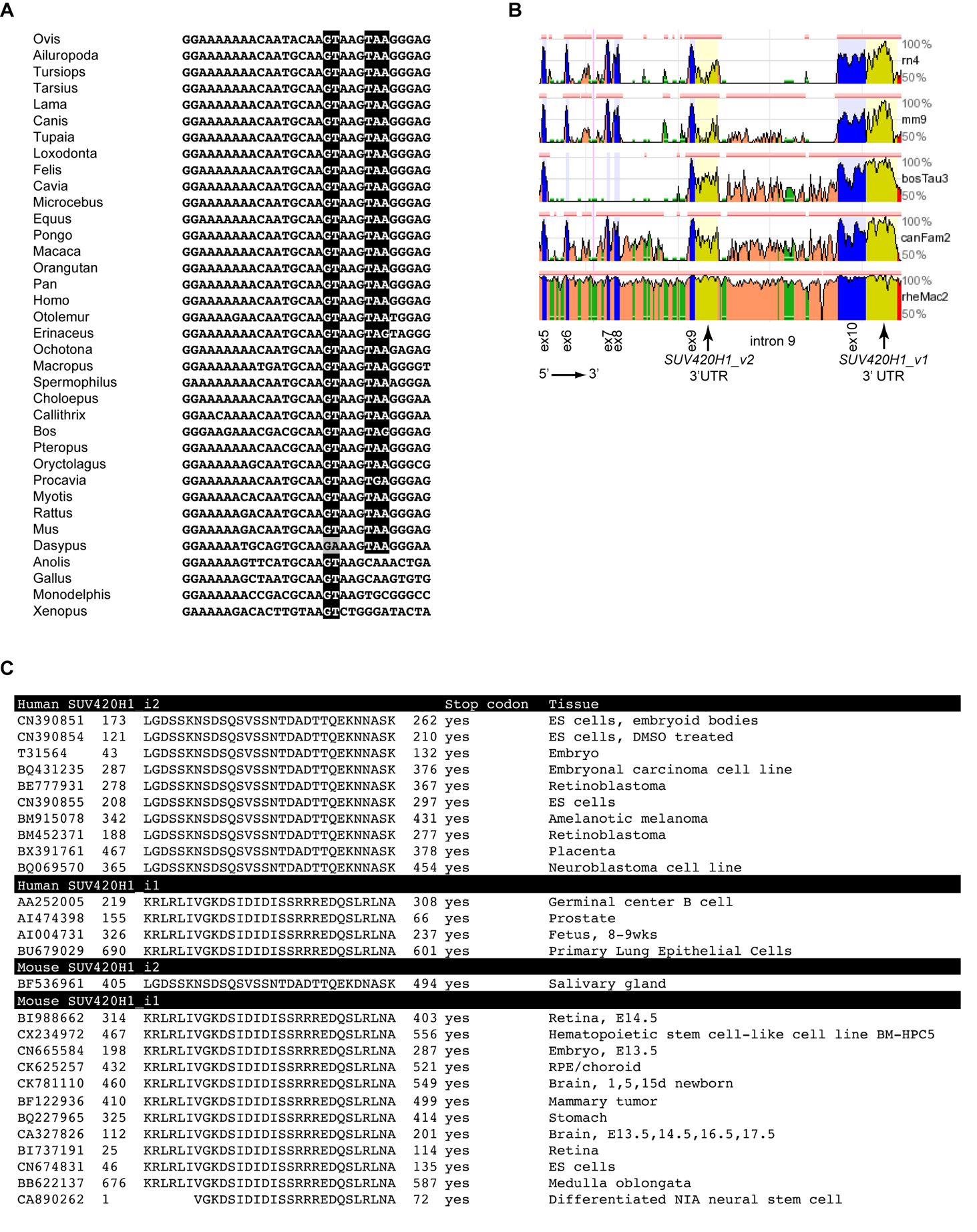

Supplement: Figure S1 — Analysis of evolutionary conservation at the SUV420H1 locus. A) Multispecies alignment of the exon 9-intron 9 junction. Alignments were carried out using genomic sequences from 36 metazoan species, all of which had the splice donor, with a single occurrence of an atypical ‘GA’ donor site. Only 4 species lacked the conserved stop codon, but each contained an in-frame stop codon that added anywhere from 4-20 amino acid residues. B) Genome alignments were carried out using the Evolutionarily Conserved Regions (ECR) browser of the NCBI DCODE package (http://ecrbrowser.dcode.org) and human SUV420H1 (chr11:67680083-67737360) as the query sequence. Output from the ECR browser is shown for a region of the human SUV420H1 gene spanning exons 5 to 10 (exons are depicted in blue, introns in pink, UTRs in yellow, repetitive elements in green, and intergenic regions in red). The sequence 3′ of exon 9, is annotated as a UTR and shows a higher degree of conservation than regions downstream of other exons (including exons 1-4, not shown). Alignments include the corresponding region from the genomes of Rattus norvegicus, Mus musculus, Bos taurus, Canis familiaris, and Rhesus macaques. C) Expressed sequence tags for the SUV420H1 isoforms. A 30aa segment at the C-terminus of each SUV420H1 isoforms was used to analyze the mouse and human components of the Expressed Sequence Tag (EST) database using tBlastn. The accession numbers and position of the coded sequence within the EST are shown. In each case, the presence of the stop codon was verified and the source tissue or cell line is indicated. (8.14 MB TIF) [file pone.0014447.s001.tif]

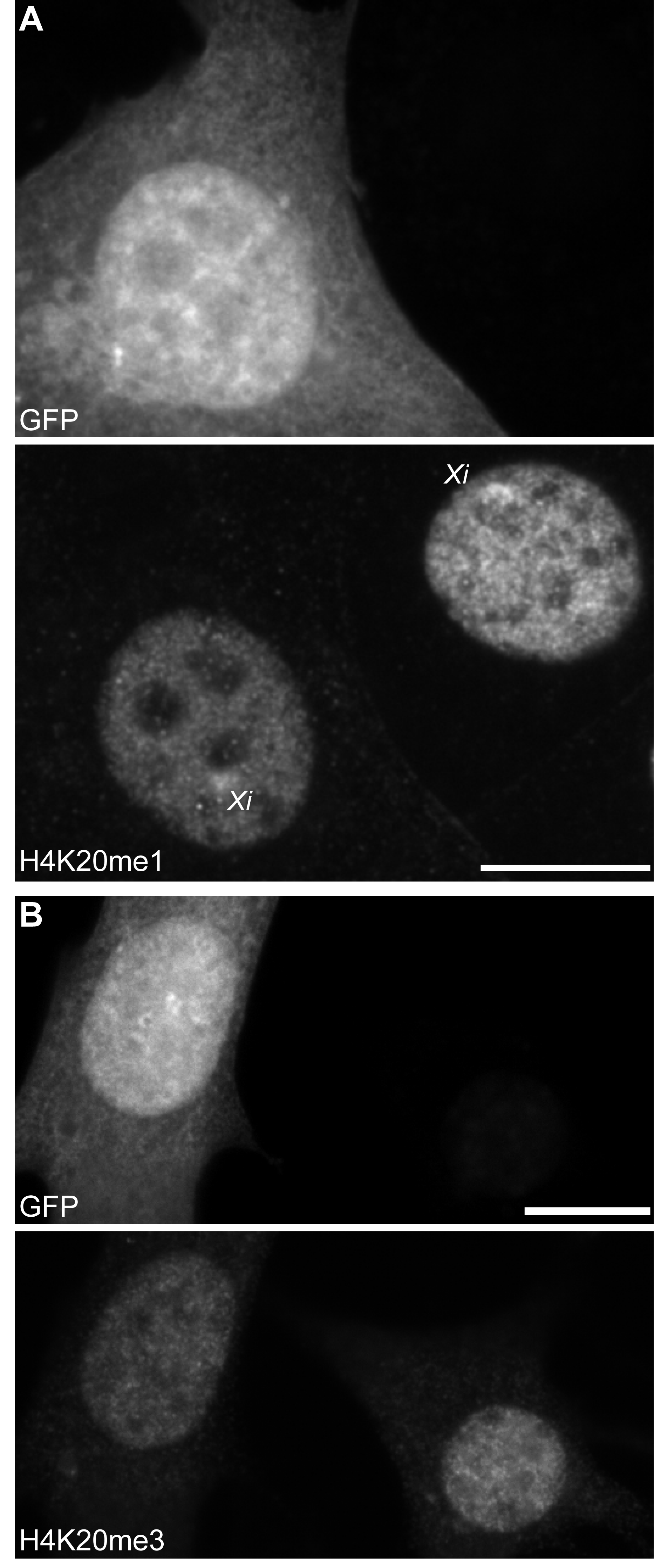

Supplement: Figure S2 — Affect of exogenous GFP expression on H4K20me1 and H4K20me3. C3H 10T1/2 cells were transfected with the pEGFP-N1 expression plasmid (Clontech) and processed for immunofluorescence using antibodies specific for H4K20me1 (A) or H4K20me3 (B). In each case, the upper panel shows a representative micrograph of adjacent cells, where the cell on the left expresses GFP. The lower panels depict staining with H4K20me1 or H4K20me3 antibodies. Enrichment of H4K20me1 on the inactive X chromosome (Xi) is indicated. Scale bar is 10 µm. (8.38 MB TIF) [file pone.0014447.s002.tif]
